# A Denoising Method for Randomly Clustered Noise in ICCD Sensing Images Based on Hypergraph Cut and Down Sampling

**DOI:** 10.3390/s17122778

**Published:** 2017-11-30

**Authors:** Meng Yang, Fei Wang, Yibin Wang, Nanning Zheng

**Affiliations:** Institute of Artificial Intelligence and Robotics, Xi’an Jiaotong University, Xi’an 710049, China; mengyang@xjtu.edu.cn (M.Y.); wybxy001@126.com (Y.W.); nnzheng@xjtu.edu.cn (N.Z.)

**Keywords:** ICCD image sensor, randomly clustered noise, hypergraph cut, principal component analysis, image denoising

## Abstract

Intensified charge-coupled device (ICCD) images are captured by ICCD sensors in extremely low-light conditions. They often contains spatially clustered noises and general filtering methods do not work well. We find that the scale of the clustered noise in ICCD sensing images is often much smaller than that of the true structural information. Then the clustered noise can be identified by properly down-sampling and then up-sampling the ICCD sensing image and comparing it to the noisy image. Based on this finding, we present a denoising algorithm to remove the randomly clustered noise in ICCD images. First, we over-segment the ICCD image into a set of flat patches, and each patch contains very little structural information. Second, we classify the patches into noisy patches and noise-free patches based on the hypergraph cut method. Then the noise-free patches are easily recovered by the general block-matching and 3D filtering (BM3D) algorithm, since they often do not contain the clustered noise. The noisy patches are recovered by subtracting the identified clustered noise from the noisy patches. After that, we could get the whole recovered ICCD image. Finally, the quality of the recovered ICCD image is further improved by diminishing the remaining sparse noise with robust principal component analysis. Experiments are conducted on a set of ICCD images and compared with four existing denoising algorithms, which shows that the proposed algorithm removes well the randomly clustered noise and preserves the true textural information in the ICCD sensing images.

## 1. Introduction

Intensified charge-coupled device (ICCD) images are captured by ICCD sensors in extremely low-light conditions [1]. The principle of ICCD sensors is summarized as follows. First, electrons obtained by photoelectric conversion are injected into many microchannel tubes with voltage. Then, each electron crashes into the wall of a tube to generate more electrons. After that, all the electrons are ejected from the microchannel tubes to the fluorescent screen. Finally, a general CCD sensor is used to capture the image on the screen. In this way, the ICCD sensor could capture images in low-light environment. An introduction of a real ICCD image sensor was given in [2].

**Figure 1:**
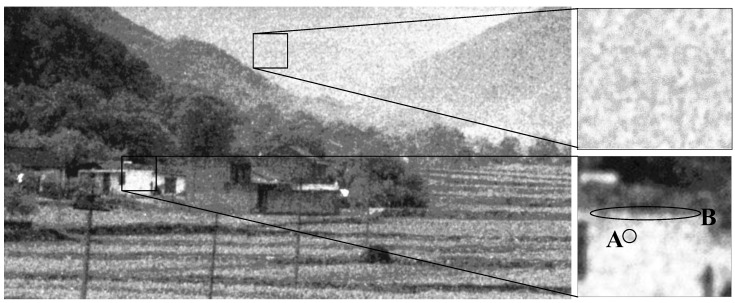
An image captured by an ICCD sensor and its noise pattern (enhanced by histogram equalization).

Although ICCD sensors have imaging abilities in extremely low-light environments, they may increase the noise of the image significantly due to the randomly generated electrons. Therefore, the noise in ICCD sensing images is more complicated than that in natural images. It generally has the following two characteristics: (a) Different from the independent and identically distributed (*i.i.d.*) noise in natural images, the noise in ICCD images is spatially clustered due to the use of microchannel tubes, which damages the structure information of the image and induces unexpected structure information. (b) Unlike general noise with a fixed pattern, e.g., natural images with raindrop or moire patterns, the noise in ICCD images appears in a randomly clustered pattern. An example of ICCD images is shown in Figure 1. It can be seen that the ICCD image induces unexpected structure information and its pattern appears random, which consequently destroys the true structure information of the image. A deep analysis of the random clustered noise has been given in [2], by comparing it with that of natural images. Indeed, this problem exists not only in ICCD images, but also in other possible scenarios, such as images captured in front of a frosted window.

In this paper, we focus on removing the randomly clustered noise in ICCD sensing images, which are captured by the ICCD sensor [2]. The noise removal problem has been well studied for *i.i.d.* noise and fixed pattern noise in the past decades [3]. However, the existing algorithms for *i.i.d*. noise generally do not work well for ICCD images, because the large scale and intensity of the clustered noise may be wrongly treated as true structure information with these algorithms. Existing algorithms for pattern noise are generally suitable for noise with fixed patterns only, such as water droplets or raindrops in natural images [4,5,6,7,8]. Other fixed pattern noise removal problems were also addressed by various solutions in the past. For example, moire pattern noise removal was studied in [9,10,11]. An algorithm for textural pattern noise removal was proposed in [12], based on a nonlocal means filter [13]. The pattern noise in document analysis systems such as stroke-like patterns and background patterns was studied in [14,15]. The researchers in [16] modeled and removed the fixed pattern noise in photonic mixture devices. The researchers in [17] proposed a framework for a video jointly corrupted by random noise and spatially correlated fixed-pattern noise. Recently, the researchers in [18] discussed the noise removal of a newly emerging pattern, i.e., the canvas patterns in digital acquisitions of paintings. One unified solution for these fixed pattern noises is self-learning-based signal decomposition [6,7,8]. In this solution, the image is first decomposed into a low-frequency component and a high-frequency component, and a dictionary is learned from the high-frequency component. Then, the feature of the noise pattern is extracted from the dictionary elements. After that, the dictionary is classified into a noise-free sub-dictionary and noise sub-dictionary by classifiers or clustering [19]. The noise-free sub-dictionary is used to reconstruct the high frequency component by sparse representation. Finally, the image is restored by combining the low-frequency component and the reconstructed high-frequency component. However, all these existing algorithms were designed for fixed pattern noises, e.g., fixed shape or characteristic noise. Thus, they may not be suitable for the randomly clustered noise in ICCD images. In [2], we studied this problem based on sparse representation. However, it often loses some textural information of the ICCD image in the smoothing process.

In this paper, we attempt to remove the randomly clustered noise in the ICCD images that are captured by ICCD sensors, while preserving their textural information. We found that the scale of the clustered noise (e.g., the noise in region A in Figure 1) is often much smaller than that of the real structural information (e.g., the edge in region B in Figure 1) in ICCD images. Based on this finding, we propose to down-sample the ICCD image based on the scale of the noise and then up-sample it. In this way, the clustered noise in the ICCD image can be estimated by calculating the residuals between the up-sampled image and the noisy one. Then, the ICCD image can be classified into noisy patches and noise-free patches based on the significance of the clustered noise. To this end, we over-segment the ICCD image into non-overlapped patches and each patch does not include obvious structural information, for which we adopt the watershed algorithm [20]. To identify noisy patches accurately, we take the segmented patches as vertices and construct the hypergraph [21] to model the relationship between the patches, then the patches are classified into noisy patches and noise-free patches with the hypergraph cut algorithm [21]. 

Based on the patch classification process, we filter the noisy patches and noise-free patches separately. For the noise-free patches, they do not include significant clustered noise. They can be easily recovered with general image filtering methods for natural images. For the noisy patches, the clustered noise can be recovered by subtracting the mean value of the residuals in each patch from the noisy one. Then the recovered ICCD image can be obtained with all these recovered patches. In the experiment, we find that the recovered ICCD image still includes some sparsely clustered noise. Thus, we further post-process the recovered ICCD image by diminishing its noise based on a robust principal component analysis. The proposed noise removal algorithm is evaluated on an ICCD image dataset and compared with four existing denoising algorithms, which demonstrates that the proposed algorithm effectively removes the randomly clustered noise in the ICCD image and preserves the textural information.

The rest of the paper is organized as follows. In Section 2 we introduce related models that are required in developing the proposed algorithm. In Section 3, we introduce the framework of the proposed algorithm. In Section 4, we present the algorithm in detail. The experiments are described in Section 5 and the conclusion is made in Section 6.

## 2. Related Models

In this section, we briefly review three related models that were used in developing the proposed algorithm, including the hypergraph and hypergraph cut [21], the spectral clustering [22,23] and the robust principal component analysis [24].

### 2.1. Hypergraph and Hypergraph Cut

The hypergraph is a mathematical model generalized from the normal graph. It uses a subset of vertices as an edge that is called a hyperedge (denoted e). One hyperedge can connect more than two vertices. Let *V* be the set of vertices, E be the set of subsets of *V* that meet $\cup_{e\in E}=V$. Then G=(V,E,ω) is called a hypergraph with vertex set *V* and edge set *E*, and each hyperedge *e* is assigned a weight ω(e). For a vertex v∈V, its vertex degree is defined as $d(v)=\sum_{\left\{e\in E|v\in e\right\}}\omega (e)$. For a hyperedge e∈E, its hyperedge degree is defined as δ(e)=|e|.

Based on the defined hypergraph, the hypergraph cut problem is described as follows. For a vertex subset S⊂V, denoting $S^{c}$ as the complement of *S*, a cut of the hypergraph G=(V,E,ω) is a division that divides *V* into *S* and $S^{c}$. If an edge e contains both elements in *S* and $S^{c}$, this edge will be cut. We define the hyperedge boundary of *S* as the set of cut hyperedges, i.e., $\partial S\equiv \left\{e\in E|e\cap S\neq \emptyset ,e\cap S^{c}\neq \emptyset \right\}$. The volume of S is defined as $volS\equiv \sum_{v\in S}d\left(v\right)$ and the volume of ∂S is defined as:

(1) $$vol\partial S\equiv \underset{e\in S}{\sum }\omega \left(e\right)\frac{\left|e\cap S\right|\left|e\cap S^{c}\right|}{\delta \left(e\right)}$$

Then the cut of the hypergraph ∂S can be obtained by solving the following optimization problem:

(2) $$\partial S=\underset{\emptyset \neq S\subset V}{\arg\min}\;vol\partial S\left(\frac{1}{volS}+\frac{1}{volS^{c}}\right)$$

Similar to the normalized cut, the minimization problem in Equation (2) is a non-deterministic polynomial problem, which can be relaxed into a real-valued optimization problem [22]. Then it can be solved with the spectral analysis algorithm in [21]. 

### 2.2. Spectral Clustering

Given a similarity matrix A, where A(i,j) represents the similarity between sample *i* and sample *j*, the Laplacian matrix is defined as $L=D^{-1/2}(D-A)D^{-1/2}$, where D is the diagonal matrix $D(i,i)=\sum_{j}A(i,j)$. Then unsupervised data clustering can be obtained by decomposing the eigenvalues of the Laplacian matrices L. One popular way to get the clustering result is to use the k-means method on the first several eigenvectors associated with the smallest non-zero eigenvalues. A common construction of the similarity matrix **A** is:

(3) A(i,j)=exp(−dis(i,j))

where dis(i,j) is defined as the distance between vertex *i* and vertex *j*. Then different similarity matrices can be obtained by constructing different dis(i,j).

### 2.3. Robust Principal Component Analysis

We consider a data matrix X, in which its rows or columns contain similar structural information as well as sparse noise. Then the matrix X can be decomposed into two matrices: one is the low rank matrix (denoted A) that represents the true structural information, the other is the sparse matrix (denoted E) that represents the noise. The decomposition of X can be achieved by solving the following optimization problem:

(4) $$\underset{A,E}{\min}\;rank\left(A\right)+\lambda {\left\|E\right\|}_{0}\;s.t.\;X=A+E$$

where ${\left||\cdot |\right|}_{0}$ is the $l^{0}$ norm, and λ is a non-negative constant value. Since minimizing the rank and $l^{0}$ norms is non-convex and non-smooth, Equation (4) is generally transformed into the following relaxed convex optimization problem:

(5) $$\underset{A,E}{\min}{\left\|A\right\|}_{*}+\lambda {\left\|E\right\|}_{1}\;s.t.\;X=A+E$$

where ${\left||\cdot |\right|}_{*}$ is the kernel norm and ${\left||\cdot |\right|}_{1}$ is the $l^{1}$ norm. The convex optimization problem in Equation (5) is called robust principal component analysis (RPCA) [24], which is an important model of low rank representation. There are many ways to solve the RPCA, such as augmented Lagrange multiplier (ALM) [25], accelerated proximal gradient [26], and dual method [26].

## 3. Framework of the Proposed Denoising Algorithm

**Figure 2:**
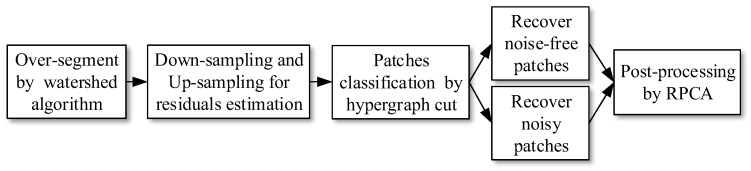
The framework of the proposed denoising algorithm for ICCD images.

The framework of the proposed denoising algorithm is shown in Figure 2. First, the ICCD image is over-segmented by the watershed algorithm [20], which could roughly segment the ICCD image into a set of flat patches. Then the ICCD image is properly down-sampled and up-sampled to extract the residuals of each patch. It is used to classify the patches into noise patches and noise-free patches based on the hypergraph cut model. After that, the noise patches and noise-free patches are filtered separately to obtain the whole recovered image. Finally, the recovered ICCD image is further post-processed with RPCA to remove the remaining sparse clustered noise in the recovered ICCD image. The details of the proposed algorithm are described in Section 4.

## 4. The Denoising Algorithm for ICCD Sensing Images

In this section, we present the proposed denoising algorithm in detail, including five steps, i.e., the over-segmentation with the watershed algorithm, the down-sampling and up-sampling for residual estimation, construction of the hyperedges and their weights, the patch classification and recovery, and the post-processing with RPCA.

### 4.1. Over-Segmentation with the Watershed Algorithm

**Figure 3:**
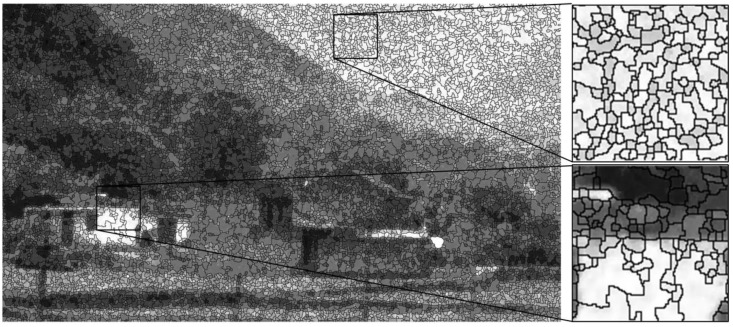
The segmentation result with watershed algorithm on an ICCD image.

First of all, we over-segmented the ICCD image into non-overlapped flat patches, where each patch does not include obvious structural information. To this end, the ICCD image should be segmented according to the textural edges of the image, for which we adopted the watershed algorithm. The result of the segmentation on an ICCD image is shown in Figure 3.

### 4.2. Down-Sampling and Up-Sampling for Residual Estimation

In Section 4.1, the ICCD image is segmented into non-overlapped flat patches. Considering that the scale of the clustered noise is often smaller than that of the true structural information in the ICCD image, we down-sampled the ICCD image at a pre-defined scale to obtain a low-resolution ICCD image. It mainly includes the true structural information. Then we could get a recovered ICCD image by filtering the low-resolution ICCD image with a general filter and up-sampling it. In the experiment, we found that the scale of the clustered noise varies in the ICCD image as well as the size of the patches. Down-sampling and up-sampling with an overly large scale value may result in serious distortion of the restored image. Thus, rather than adopting a fixed scale in the down-sampling process, we pre-defined the scale value for each patch separately as follows.

First, we defined a main direction vector for each patch by searching the two pixels that have the longest line in each patch. In the experiment, to improve the robustness of this process, we search the 5% longest lines in the patch and define the main direction vector by averaging all these vectors. The length of the main direction vector is defined as the length of the patch. Similarly, we find the line that is perpendicular to the main direction and define its length as the width of the patch. We define the patch scale as follows:

(6) $$Scale=\frac{width}{length}\cdot width$$

Then, we classify patches into several classes according to their scale values, and take the corresponding scale as the sampling scale of the patches in each class in the down-sampling and up-sampling process, rather than adopting the fixed scale values for them. The process of the down-sampling and up-sampling is concluded as follows.

(a) Calculate the scale of each patch, and then classify them into several classes according to their scale values with the k-means method [27].
(b) For each class, determine the mean of patch scales as the sampling scale in the down-sampling process.
(c) Denoise the image after each down-sampling with the general block-matching and 3D filtering (BM3D) [28] algorithm;
(d) Up-sample each low-resolution image to the original size to obtain a recovered ICCD image.

After getting the recovered image, the residuals of each patch can be obtained by comparing the patches in the recovered image and the noisy ICCD image as follows:

(7) $$RD=\frac{1}{N}\overset{N}{\underset{l=1}{\sum }}{\left(x_{R}^{\left(l\right)}-x_{O}^{\left(l\right)}\right)}^{2}$$

where N is the number of pixels in the current patch, $x_{R}^{\left(l\right)}$ and $x_{O}^{\left(l\right)}$ are the gray value of the *l*-th pixel in the restored image and noisy image for the current patch. Generally, a patch with clustered noise is given a larger residual value and a patch without clustered noise is given a smaller residual value. Thus, the residual value can be used to classify the patches into noisy patches and noise-free patches, which will be introduced in the next two sections.

### 4.3. Construction of Hyperedges and Their Weights

We classified the segmented patches into noisy patches and noise-free patches based on the estimated residuals in Section 4.2. Considering that the noisy patches of neighboring areas have similar gray values, we can take the gray value and position information into account to help identify the noisy patches more accurately together with the residual information. Unfortunately, the relationship between the patches becomes complex due to the coupling of different information (i.e., the gray value and position information). To address this problem, we used the hypergraph to model the relationship between the segmented patches. First of all, we took the segmented patches as vertices and constructed hypergraphs to model the relationship between the patches. The vertices that have similar attributes are connected by a hyperedge. We adopt the spectral clustering method to construct hyperedges, and each hyperedge is assigned a weight according to the distance between the vertices. Then the complex relationship between patches is described by all the hyperedges, and the binary classification problem can be converted to a graph cut problem. The detailed process is described as follows.

**A. Construction of a Hyperedge Based on Spectral Clustering**

We first constructed the distance of two patches in Equation (3) with their residual values as:

(8) $$dis^{R}\left(i,j\right)=|RD_{i}-RD_{j}|/\sigma^{R}$$

where $RD_{i}$ and $RD_{j}$ are the RD value of the *i*-th and the *j*-th patch, respectively, and $\sigma^{R}$ is the standard deviation of $\left\{|RD_{i}-RD_{j}||i,j\in V\right\}$. The obtained distance values $dis^{R}\left(i,j\right)$ in Equation (8) are further normalized to [0, 1]. Then the similarity matrix A in Equation (3) can be constructed with $dis^{R}\left(i,j\right)$. Then the patches can be divided into *m* different classes based on the spectral clustering method, and the *m* classes correspond to a set of constructed hyperedges. The parameter *m* will be determined by the experiment in Section 5.1. The obtained hyperedges do not constitute a hypergraph since they are still disconnected. We repeated the clustering method *n* times to obtain multiple sets of constructed hyperedges. The parameter *n* will be determined by the experiment in Section 5.1. All the hyperedges can be used to construct a hypergraph.

To improve the robustness of the hypergraph, we additionally added other information from the patches into the hypergraph besides the residual values. Considering that neighboring patches generally have similar gray values, we adopted the position and gray-scale information of the patches to further construct three similarity matrices AG, AP, AM, with their distances defined as:

(9) $$dis^{G}\left(i,j\right)=\left(1-\alpha \right)dis^{R}\left(i,j\right)+\alpha ||g_{i}-g_{j}|{|}_{2}/\sigma^{G}$$

(10) $$dis^{P}\left(i,j\right)=\left(1-\alpha \right)dis^{R}\left(i,j\right)+\alpha {\left[{\left(x_{i}-x_{j}\right)}^{2}+{\left(y_{i}-y_{j}\right)}^{2}\right]}^{1/2}/\sigma^{P}$$

(11) $$dis^{M}\left(i,j\right)=dis^{G}\left(i,j\right)+dis^{P}\left(i,j\right)-dis^{R}\left(i,j\right)$$

where α is a constant parameter, $g_{i}$ and $g_{j}$ are the gray histogram of the *i*-th and the *j*-th patches, respectively, ${\left||\cdot |\right|}_{0}$ is the $l^{2}$ norm, and $\sigma^{G}$ is the standard deviation of $\left\{||g_{i}-g_{j}|{|}_{2}|i,j\in V\right\}$; $x_{i}$, $y_{i}$ and $x_{j}$, $y_{j}$ are the center coordinates of the *i*-th and the *j*-th patches, respectively, $\sigma^{P}$ is the standard deviation of $\text{\{}{\left[{\left(x_{i}-x_{j}\right)}^{2}+{\left(y_{i}-y_{j}\right)}^{2}\right]}^{1/2}|i,j\in V\text{\}}$. Similar to Equation (8), the distance values in Equations (9)–(11) are further normalized to [0, 1]. The parameters α will be determined together with *m* and *n* by the experiment in Section 5.1.

**B. Calculation of the Hyperedge Weight**

After the construction of hyperedges, we need to assign weights to each hyperedge. The weight of a hypergraph should have the following characteristics. The larger the weight value, the higher the degree of similarity in the hyperedge, and the lower the degree of similarity to the vertices in other hyperedges. We define the weight of a hyperedge as follows:

(12) $$\omega \left(e\right)=c\cdot \sum_{v\in e,u\notin e}dis\left(v,u\right)/\sum_{v,v^{\prime }\in e}dis\left(v,v^{\prime }\right)$$

where dis(⋅,⋅) is calculated in Equations (8), (9), (10) or (11) for the four similarity matrices A, AP, AG, and AM, respectively, and c is the normalization factor such that $\sum_{e\in E}\omega (e)=1$.

### 4.4. Patch Classification and Recovery

Based on the constructed hypergraph in Section 4.3, we can classify the patches into noisy patches and noise-free patches based on the hypergraph cut method in Section 2.1.

Then we recovered the noisy patches and noise-free patches, separately. Each noise-free patch can be easily recovered by filtering the patch with a general filter, since no clustered noise exists in the patch. We generally adopt the BM3D algorithm [28] for this purpose. Each noisy patch can be recovered by subtracting the associated residual value from the noisy patch. We formalize the recovery process as follows:

(13) $$x_{I}=\left(1+coef\cdot RD\right)x_{O}$$

where $x_{I}$, $x_{O}$ are the gray values of the pixels in the recovered patch and the noisy patch, respectively. The variable coef is defined as the inverse of the maximum gray value in the noisy patch as $coef={\left(max\left\{x_{O}\right\}\right)}^{-1}$, where $\left\{x_{O}\right\}$ is the set of gray values of the pixels in a noisy patch. Then we further used the BM3D algorithm on the recovered patch. With all the recovered noisy patches and noise-free patches, we were able to obtain the whole recovered ICCD image.

### 4.5. Post-Processing by RPCA

In the experiment, we found that sparse clustered noise still existed in the recovered ICCD image. Thus, we further post-processed the image with the RPCA described in Section 2.3. Firstly, we constructed the data matrix X as follows. We decomposed the image into overlapping blocks of size 64 × 64 with a step of 16 × 16, and then classified the image blocks into groups with spectral clustering. Each group of blocks forms a low rank matrix, where the gray value of pixels in an image block corresponds to a column in matrix X. Then the low rank matrix A can be obtained by solving the problem in (5) and the image blocks can be recovered from matrix A. Finally, the recovered ICCD image can be obtained by averaging the overlapped block parts. Secondly, according to the ALM algorithm in (5) and [25], we can obtain the corresponding recovery matrix A of each group, then the *j*-th column of matrix A corresponds to the *j*-th restored image block in the group. Finally, since the process of image decomposition allows overlapping image blocks, we take the average of the overlapping parts to get the whole restored image.

The parameter λ in the optimization problem in (5) plays an important role in determining matrix A. Specifically, the smaller the parameter λ, the smoother the recovered image, and more details of the image will be eliminated. The larger the parameter λ, the more details will be preserved. However, it cannot well eliminate the sparse noise in the image. Thus, rather than fixing the parameter λ value, we adaptively adjusted it according to the histogram variance of oriented gradient (HVOG) value [2] of each group. In this way, it can keep the structure information and eliminate the noise well. The parameter λ for each group of blocks is defined as:

(14) $$\lambda =\exp(-1/\gamma_{0}\cdot HVOG)$$

where HVOG is the average value of the normalized *HVOG* value of the blocks in each group, and $\gamma_{0}$ is a constant parameter $\gamma_{0}$ = 10.

## 5. Experiments and Analysis

In this section, we verify the performance of the proposed method on a dataset of real ICCD images, which were captured by an ICCD camera [2] in extremely low-level light conditions. It is worth noting that since the ground truth of the real ICCD images is not available, we mainly evaluate the subjective performance of the proposed algorithm in the following. We first determine the relevant parameters in constructing a hypergraph by experiment in Section 5.1, and verify the effectiveness of the hypergraph cut in the filtering process in Section 5.2 by experiment. After that, we verify the performance of the proposed denoising algorithm on a set of real ICCD images and compare it with four existing denoising algorithms in Section 5.3. We also test the effectiveness of the RPCA for denoising in the proposed algorithm in Section 5.4.

### 5.1. Parameter Determination in Constructing the Hypergraph

We determined the parameters in constructing the hypergraph on a set of ICCD images with simulated noise [2]. We performed the hypergraph cut with different parameters in the proposed algorithm to obtain the average objective quality (PSNR) values for the dataset. Table 1 shows part of the objective results at different parameter values, where n is the number of times of clustering, m is the number of clusters at each time, α is the weight parameter in Equations (9)–(11), and PSNR is the average PSNR value of the processed dataset. It can be seen that the values of the parameters n, m and α have a slight impact on the results for the results in Table 1. This verifies the high robustness of the hypergraph. We determine the parameters as n = 3, m = 2, α = 0.5 based on Table 1, at which the PSNR result is maximum.

### 5.2. Verify the Effectiveness of the Hypergraph Cut

**Figure 4:**
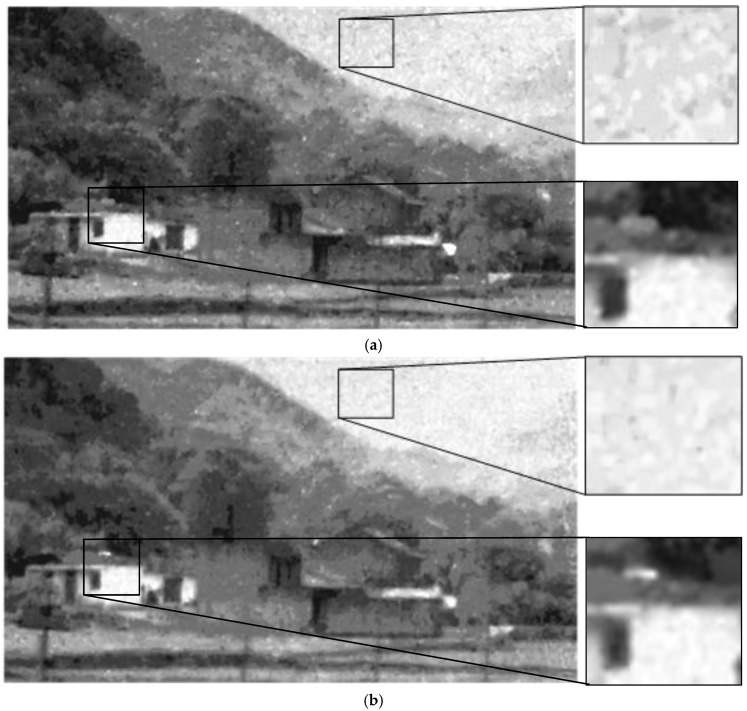
Verify the effectiveness of the hypergraph cut. (**a**) the filtering result based on spectral clustering; (**b**) the filtering result based on the hypergraph cut.

The hypergraph cut method is used for patch classification in the filtering algorithm. In order to test its effectiveness, we replace it with a general classification method, i.e., spectral clustering, and compare the performance of the two filtering algorithms on the example in Figure 4. Figure 4a shows the filtering result based on the spectral clustering and Figure 4b shows the result based on the hypergraph cut. It can be seen that the spectral-clustering-based filtering method does not remove the clustered noise well. This is mainly because the regions with clustered noise are not fully and correctly recognized in the patch classification process. By comparison, the hypergraph-cut-based filtering method performs better.

### 5.3. Verify the Performance of the Proposed Denoising Algorithm

We then tested the performance of the proposed ICCD image denoising algorithm on a set of real ICCD images. To our knowledge, in the past there have been few denoising algorithms that have considered randomly clustered noise, except for [2]. Thus, we mainly compare with the algorithm in [2] and three general denoising algorithms of natural images, including BM3D [28], the K-Singular-value-decomposition (K-SVD) based denoising method [29] and the Bayes least squares-Gaussian scale mixtures (BLS-GSM) denoising algorithm [30]. Since the ground truth of the real ICCD images is not available, we mainly show the subjective results of proposed method.

**Figure 5:**
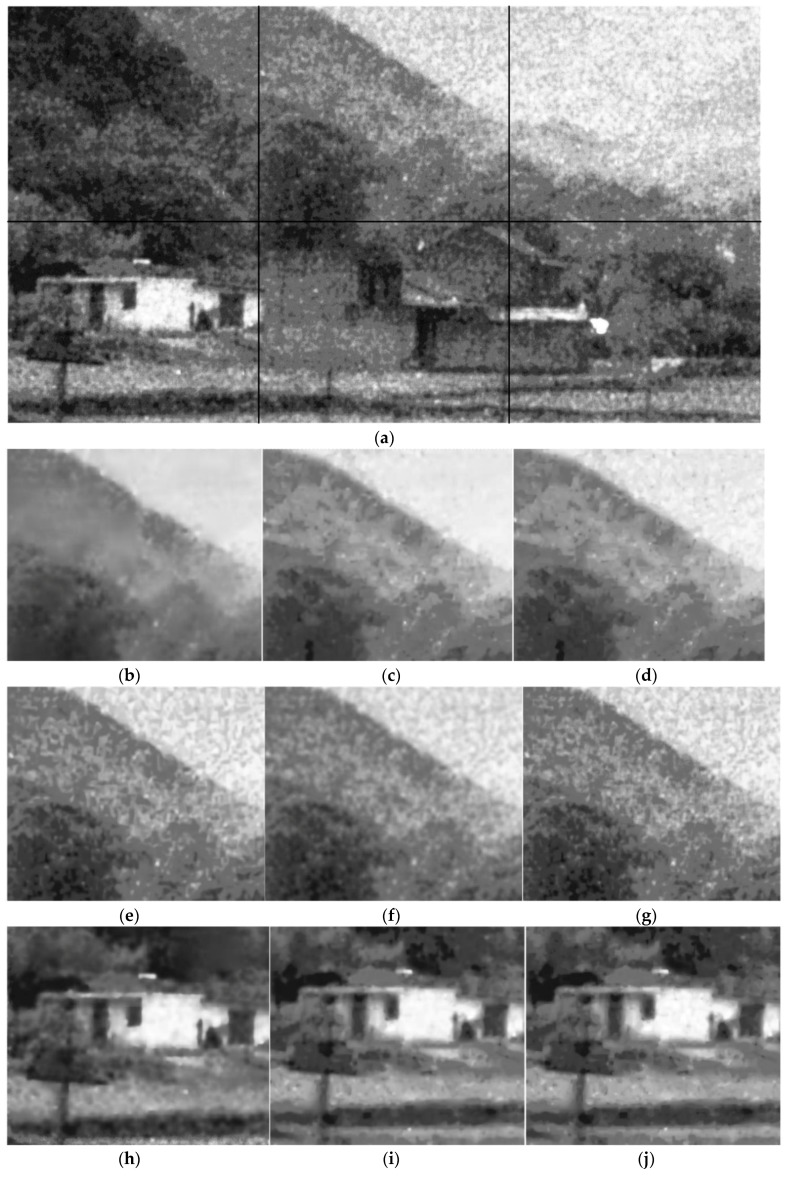

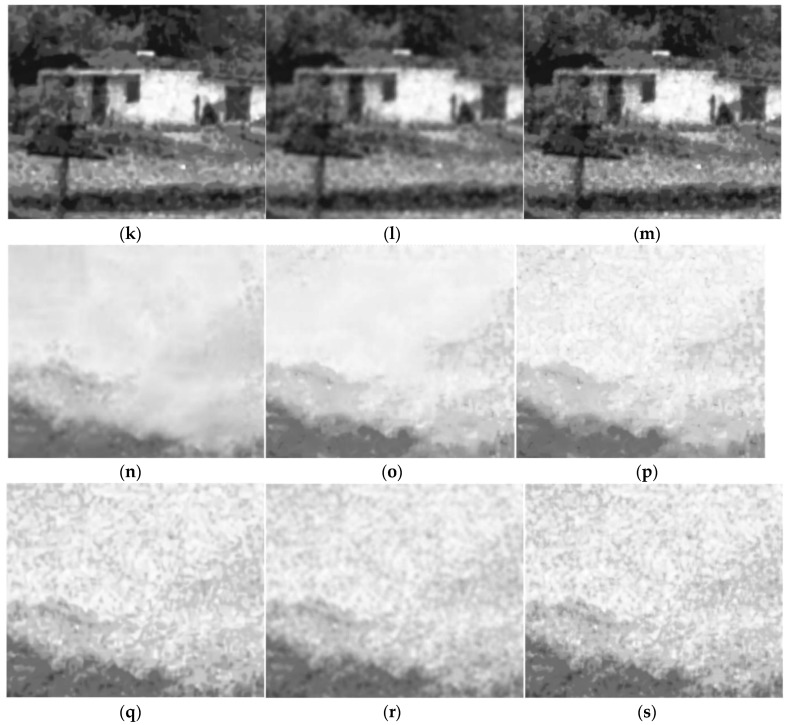
The subjective results of the proposed algorithm and other baselines. (**a**) the patches in the noisy ICCD image; (**b**,**h**,**n**) the result of the denoising algorithm in [2]; (**c**,**i**,**o**) the result of the proposed algorithm; (**d**,**j**,**p**) the result without the RPCA in Section 4.5; (**e**,**k**,**q**) the results of BM3D; (**f**,**l**,**r**) the result of K-SVD; (**g**,**m**,**s**) the results of BLS-GSM.

**Figure 6:**
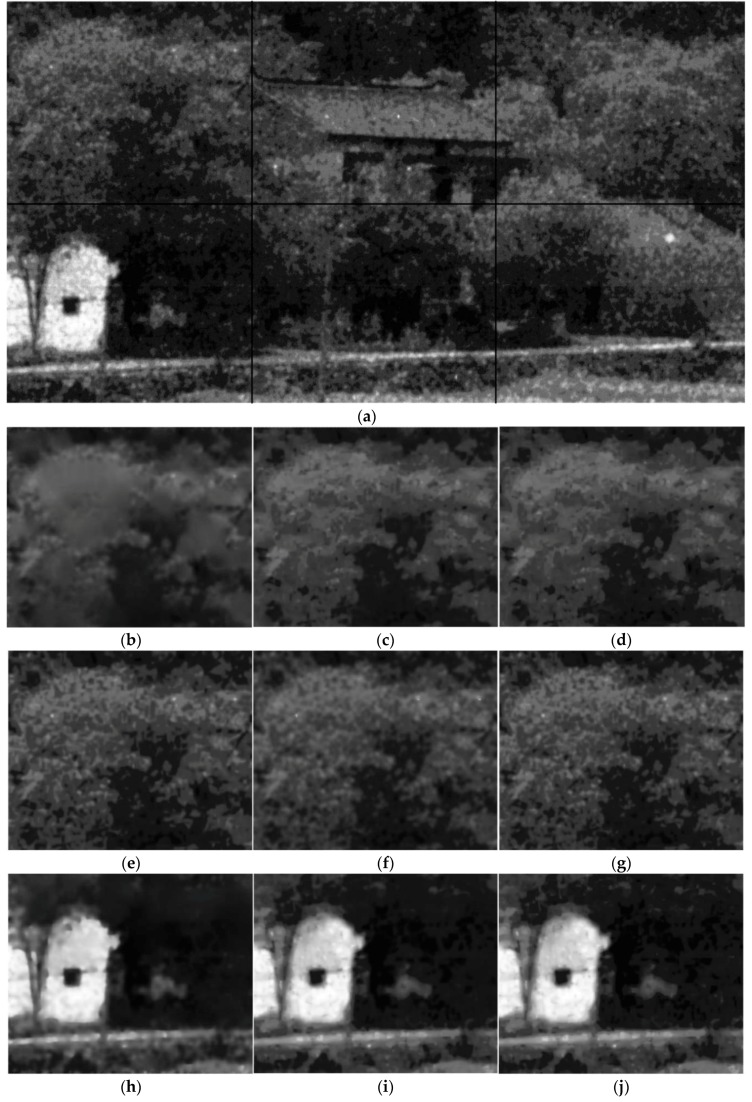

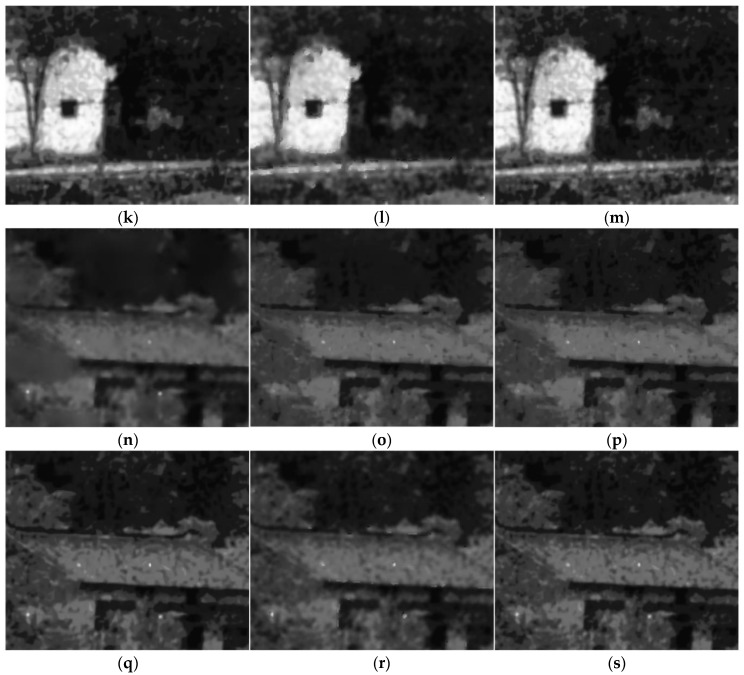
The subjective results of the proposed algorithm and other baselines. (**a**) the patches in the noisy ICCD image; (**b**,**h**,**n**) the result of the denoising algorithm in [2]; (**c**,**i**,**o**) the result of the proposed algorithm; (**d**,**j**,**p**) the result without the RPCA in Section 4.5; (**e**,**k**,**q**) the results of BM3D; (**f**,**l**,**r**) the result of K-SVD; (**g**,**m**,**s**) the results of BLS-GSM.

**Figure 7:**
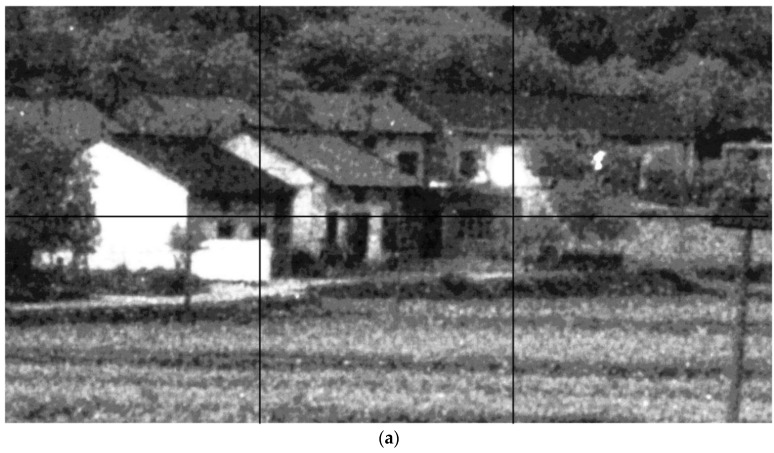

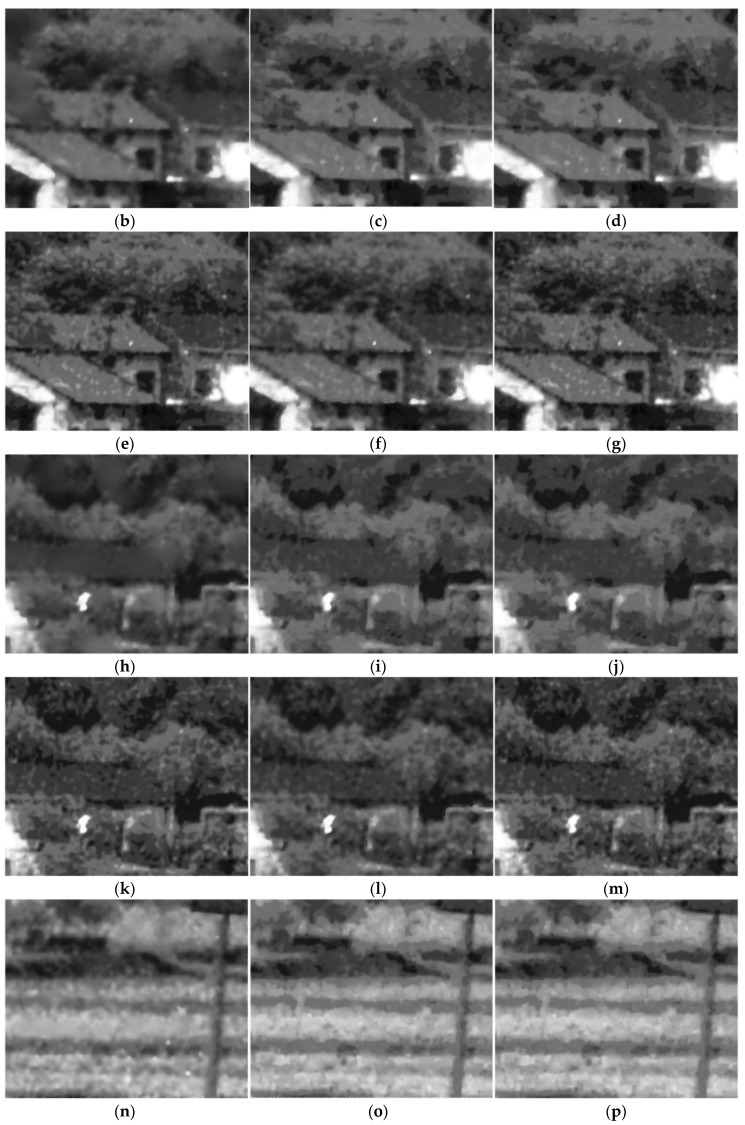

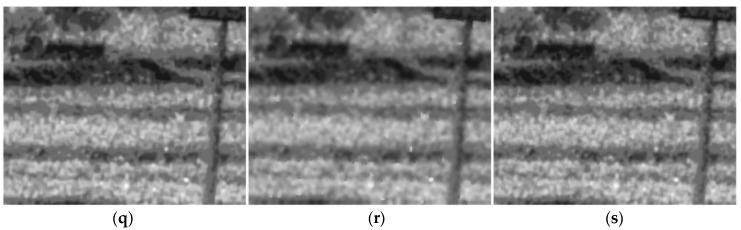
The subjective results of the proposed algorithm and other baselines. (**a**) the patches in the noisy ICCD image; (**b**,**h**,**n**) the result of the denoising algorithm in [2]; (**c**,**i**,**o**) the result of the proposed algorithm; (**d**,**j**,**p**) the result without the RPCA in Section 4.5; (**e**,**k**,**q**) the results of BM3D; (**f**,**l**,**r**) the result of K-SVD; (**g**,**m**,**s**) the results of BLS-GSM.

Part of the results are shown in Figure 5, Figure 6 and Figure 7. It can be seen that both the proposed algorithm and the algorithm in [2] could effectively remove the randomly clustered noise. By comparison, the general filtering methods BM3D and BLS-GSM do not work well for randomly clustered noise. More seriously, the clustered noise in the ICCD images is even enhanced with these methods. This is because the clustered noise is often wrongly treated as true structural information with these methods. The K-SVD-based denoising method could partly remove the clustered noise in the ICCD images. However, it may induce significant distortion in the structure regions. We further compare the performance of the proposed algorithm with that of [2]. It can be seen that the images filtered with [2] are often much smoother, however, some textural details are lost in the filtered images. By comparison, the proposed algorithm can retain detailed information, e.g., the leaf pattern of the trees in Figure 6.

### 5.4. Verify the Effectiveness of the RPCA in the Denoising Process

**Figure 8:**
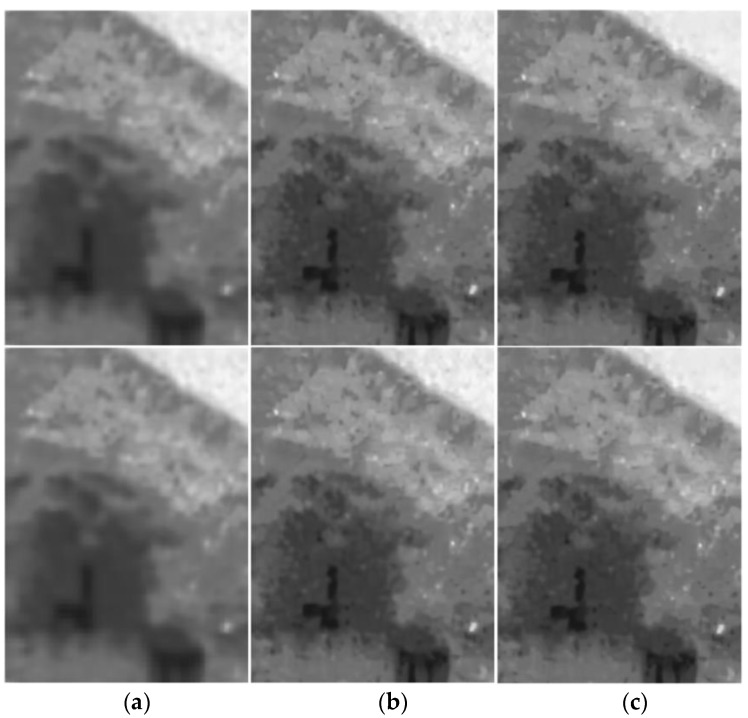
Verify the effectiveness of the RPCA in the filtering process. (**a**) the results of the RPCA with λ = 0.1; (**b**) the results of the RPCA with λ = 0.9; (**c**) the results of the RPCA with the adaptive parameters.

We further verified the effectiveness of the RPCA in Section 4.5 in the filtering process by comparing the results of the filtering algorithm with different parameters λ in the RPCA. Some results are shown in Figure 8, where Figure 8a shows the result with the parameter λ = 0.1. Figure 8b shows the result with λ = 0.9. Figure 8c is the result of the RPCA with the adaptive parameters in Section 4.5. It can be seen from the results that the smaller the parameter λ, the smoother the result and the more textural details are eliminated in the filtered image. On the contrary, the larger the parameter λ, the more textural details are retained. However, the sparse noise cannot be well eliminated. By comparison, the described RPCA with an adaptive λ value makes a good balance for this problem, which can be seen from the results in Figure 8c.

### 5.5. Time Complexity of the Proposed Algorithm

In this section, we present the time complexity of the proposed algorithm (with or without the RPCA) and compare it with that of the algorithm in [2]. The size of the tested images was fixed as 1545 × 840, and the algorithms were implemented on a platform of i5-2400 CPU, 16G-RAM, and Windows 10 OS (DELL, Shanghai, China), and the algorithms were carried out on MATLAB 2014b. The average results are recorded in Table 2. It can be seen that the complexity of the proposed algorithm mainly comes from the RPCA process in Section 4.5. When the RPCA is inactivated, the proposed algorithm is 10 times faster than the algorithm in [2]. Even though the RPCA is activated, the proposed algorithm still saves a runtime of over 30% compared with that of [2].

## 6. Conclusions

In this paper, we designed a filtering algorithm to remove the clustered noise in ICCD images and preserve the true structure information. The experimental results verified the effectiveness of the proposed algorithm by comparing it with four existing denoising algorithms.

## Figures and Tables

**Table 1:** The average objective results at different parameter values in the hypergraph cut.

n	m	α	PSNR (db)	n	m	α	PSNR (db)
2	2	0.1	73.19995	3	2	0.1	73.21281
2	2	0.2	73.18212	3	2	0.2	72.95117
2	2	0.3	73.11725	3	2	0.3	73.19301
2	2	0.4	73.19670	3	2	0.4	73.28845
2	2	0.5	73.11630	3	2	0.5	73.42306
2	3	0.1	73.28173	3	3	0.1	73.04053
2	3	0.2	73.18228	3	3	0.2	73.20538
2	3	0.3	73.32812	3	3	0.3	72.88833
2	3	0.4	73.22885	3	3	0.4	73.32938
2	3	0.5	73.14801	3	3	0.5	73.19522
2	4	0.1	73.04365	3	4	0.1	73.13615
2	4	0.2	73.11540	3	4	0.2	73.19505
2	4	0.3	73.34357	3	4	0.3	72.81382
2	4	0.4	73.12557	3	4	0.4	73.14337
2	4	0.5	73.20798	3	4	0.5	72.86815
2	5	0.1	73.11540	3	5	0.1	73.19301
2	5	0.2	73.17220	3	5	0.2	73.22224
2	5	0.3	73.03383	3	5	0.3	72.78663
2	5	0.4	73.18219	3	5	0.4	73.15062
2	5	0.5	73.21231	3	5	0.5	73.17858
4	2	0.1	73.20403	5	2	0.1	73.17788
4	2	0.2	73.17530	5	2	0.2	73.19318
4	2	0.3	73.03849	5	2	0.3	73.13893
4	2	0.4	73.21268	5	2	0.4	73.03510
4	2	0.5	72.79888	5	2	0.5	73.13128
4	3	0.1	73.19837	5	3	0.1	73.14298
4	3	0.2	73.02988	5	3	0.2	73.14298
4	3	0.3	73.20614	5	3	0.3	73.07012
4	3	0.4	73.21402	5	3	0.4	73.20198
4	3	0.5	73.11114	5	3	0.5	73.13739
4	4	0.1	73.34665	5	4	0.1	73.19715
4	4	0.2	73.23437	5	4	0.2	73.08766
4	4	0.3	73.17264	5	4	0.3	73.03212
4	4	0.4	73.17855	5	4	0.4	73.11872
4	4	0.5	73.15255	5	4	0.5	73.0394
4	5	0.1	73.16033	5	5	0.1	73.16033
4	5	0.2	73.22001	5	5	0.2	73.19837
4	5	0.3	73.19912	5	5	0.3	73.14524
4	5	0.4	73.30515	5	5	0.4	73.00673
4	5	0.5	73.13857	5	5	0.5	73.24143

**Table 2:** The average runtime of the algorithms (min).

	Average Runtime
The algorithm in [2]	86.3
The proposed algorithm without the RPCA	5.4
The proposed algorithm with the RPCA	57.2
